# Activation of Toll-Like Receptor 3 Impairs the Dengue Virus Serotype 2 Replication through Induction of IFN-β in Cultured Hepatoma Cells

**DOI:** 10.1371/journal.pone.0023346

**Published:** 2011-08-04

**Authors:** Zhaoduan Liang, Siyu Wu, Yuye Li, Li He, Minhao Wu, Lifang Jiang, Lianqiang Feng, Ping Zhang, Xi Huang

**Affiliations:** 1 Department of Immunology, Institute of Immunology, Institute of Human Virology, Zhongshan School of Medicine, Sun Yat-sen University, Guangzhou, China; 2 Key Laboratory of Tropical Diseases Control (Sun Yat-sen University), Ministry of Education, Guangzhou, China; 3 Department of Microbiology, Zhongshan School of Medicine, Sun Yat-sen University, Guangzhou, China; University of Hong Kong, Hong Kong

## Abstract

Toll-like receptors (TLRs) play an important role in innate immunity against invading pathogens. Although TLR signaling has been indicated to protect cells from infection of several viruses, the role of TLRs in Dengue virus (DENV) replication is still unclear. In the present study, we examined the replication of DENV serotype 2 (DENV2) by challenging hepatoma cells HepG2 with different TLR ligands. Activation of TLR3 showed an antiviral effect, while pretreatment of other TLR ligands (including TLR1/2, TLR2/6, TLR4, TLR5 or TLR7/8) did not show a significant effect. TLR3 ligand poly(I∶C) treatment prior to viral infection or simultaneously, but not post-treatment, significantly down-regulated virus replication. Pretreatment with poly(I∶C) reduced viral mRNA expression and viral staining positive cells, accompanying an induction of the type I interferon (IFN-β) and type III IFN (IL-28A/B). Intriguingly, neutralization of IFN-β alone successfully restored the poly(I∶C)-inhibited replication of DENV2. The poly(I∶C)-mediated effects, including IFN induction and DENV2 suppression, were significantly reversed by IKK inhibitor, further suggesting that IFN-β is the dominant factor involved in the poly(I∶C) mediated antiviral effect. Our study presented the first evidence to show that activation of TLR3 is effective in blocking DENV2 replication via IFN-β, providing an experimental clue that poly(I∶C) may be a promising immunomodulatory agent against DENV infection and might be applicable for clinical prevention.

## Introduction

Dengue virus (DENV), a group of prevalent arthropod-borne viruses, belongs to the Flavirvirus genus of the family *Flaviviridae*. There are four related but distinct serotype viruses (DENV-1 to 4) [1]. While most DENV infections are asymptomatic or cause a self-limited Dengue fever (DF), some infections lead to severe and potentially lethal diseases, such as Dengue hemorrhagic fever (DHF) and Dengue shock syndrome (DSS) [1], [2]. Dengue becomes a leading infectious disease with more than 50 million cases of DF, 500 thousand cases of DHF and 20 thousand deaths each year world wide [1].

So far, DENV pathogenesis and host immune response to virus infection are largely unclear, and no effective vaccines or antiviral drugs are available for Dengue diseases. Given innate immunity especially interferons (IFNs) plays an important role in limiting viral replication during DENV primary infection, amplifying the innate immune response might be an effective strategy to defend DENV infection. There are three type IFNs, the type I IFNs, including 13 IFN-αs and a single IFN-β, IFN-ω, IFN-ε or IFN-κ, are well-known as antiviral IFNs [3]. The type I IFNs are broadly expressed in different cell types, including lymphocytes, macrophages, fibroblasts, endothelial cells, hepatoma cells and so on. The type II IFN-γ, also known as immune IFN, is expressed in natural killer (NK) or activated T cells. The type III IFN family comprises three subtypes, i.e. IL-28A/B and IL-29, which are co-produced with IFN-β and share many functional characteristics with type I IFNs [4]. After synthesis and secretion, IFNs bind to cell surface receptors and activate downstream signaling pathways via Janus kinase (JAK), tyrosine kinase (TYK) 2, signal transducers and activators of transcription (STAT) 1/2 and interferon regulatory factor (IRF) 9, resulting in an induction of more than 300 interferon stimulated genes (ISGs) [3].

The type I and II IFNs have been showed to restrict the propagation of DENV in both *in vitro* and *in vivo* models. In *in vitro* cultured cells, pretreatment with either type I IFNs (IFN-α,-β) or type II IFN (IFN-γ) were effective in inhibiting DENV infection [5]. And in *in vivo* murine model, the deficiency of IFN-α, -β, -γ receptors led to a significantly higher lethality upon intraperitoneal inoculation of DENV2 [6]. Intriguingly, a recent report indicated that the absence of type I IFN expression in DENV2 infected human monocyte-derived dendritic cells was the major reason that DC cells failed to prime T cells [7]. In terms of type III IFN, its expression pattern and role in DENV infection have not been investigated yet.

IFNs and other proinflammatory cytokines are rapidly induced by innate immune system in responding to pathogen invasion. The innate signal is originated from recognition of pathogen-associated molecular patterns (PAMPs) on pathogens by pattern recognition receptors (PRRs). For example, the PRRs to recognize DENV PAMPs include both endosomally-localized Toll-like receptors (TLR) 3, 7, 8 and cytoplamsic RIG-I/MDA5 [8], [9], [10]. Then, the activated signal is transmitted through adaptor proteins including myeloid differentiation primary response gene 88 (MyD88), Toll/IL-1 receptor domain-containing adapter-inducing interferon-β (TRIF) or interferon-beta promoter stimulator (IPS)-1. Subsequently, downstream phosphorylation cascade of the IκB kinase family (IKK-α,-β,-ε) is activated, resulting in an induction of IFNs and cytokines [3].

An important family of PRRs, TLRs, includes 10 members in humans. TLRs are responsible for detecting PAMPs of various pathogens, such as bacterial LPS (TLR4), flagellin (TLR5), unmethylated DNA (TLR9); viral dsRNA(TLR3) or ssRNA (TLR7/8), etc [11], [12], [13]. TLRs are expressed in both immune cells and non-immune cells, such as epithelial cells and hepatoma cells [12], [13]. Most TLRs signal through adaptor MyD88, except that TLR3 signals through TRIF [12]. Activation of the innate immune signaling by TLR ligands has been broadly utilized for the prevention and therapy of infectious diseases [11], [13]. So far, the protective effects of TLRs in cultured cells or animal models against a variety of viruses have been well documented. In both *in vivo* murine models and *in vitro* cultured cells, HBV replication was inhibited by TLR2, 3, 4, 5, 7, 9 agonists in a type I IFN-dependent manner [14], [15], [16]. Treatment with TLR3, 5, 7, 9 ligands significantly inhibited Herpes virus type 2 (HSV-2) replication in mice model or primary genital epithelial cells [17], [18]. And stimulation with TLR3 ligands inhibited HIV amplification in dendritic cells and macrophages [19], [20], HCV replication in human heptocytes [21], replication of influenza virus [22] and respiratory syncytial virus (RSV) [23], etc. Regarding to the effect of TLR activation on the DENV replication, only a few studies on the TLR4 activation have been reported while their conclusions were illusive. One group found that LPS inhibited DENV infection in monocytes/macrophage by blocking virus entry [24], while another group found that LPS pretreatment did not significantly reduce DENV replication in HepG2 cells [25]. Interestingly, DENV was found to counter the TLR activating pathway, further implying that TLR signaling might have some roles in blocking DENV infection [26].

The present study aimed to investigate the role of innate immune response in non-immune cells but actively infected by DENV. Our approach was to screen the effect of individual TLR ligands on DENV serotype 2 (New Guinea C strain, NGC) replication in hepatoma cells HepG2, a commonly-used DENV permissive cell line [27] with most TLR genes expressed [14], [28]. And we found that these TLR ligands play differential roles in the DENV2 infection. IFN-β induced by poly(I∶C) was identified to be the dominant antiviral agent. For the first time, we showed that the protective role of TLR3 activation on DENV replication was dependent on the time of treatment. These results provide substantial evidence that poly(I∶C) might be a promising prevention agent against Dengue diseases.

## Results

### TLR ligands played differential roles in DENV2 replication

**Figure 1:**
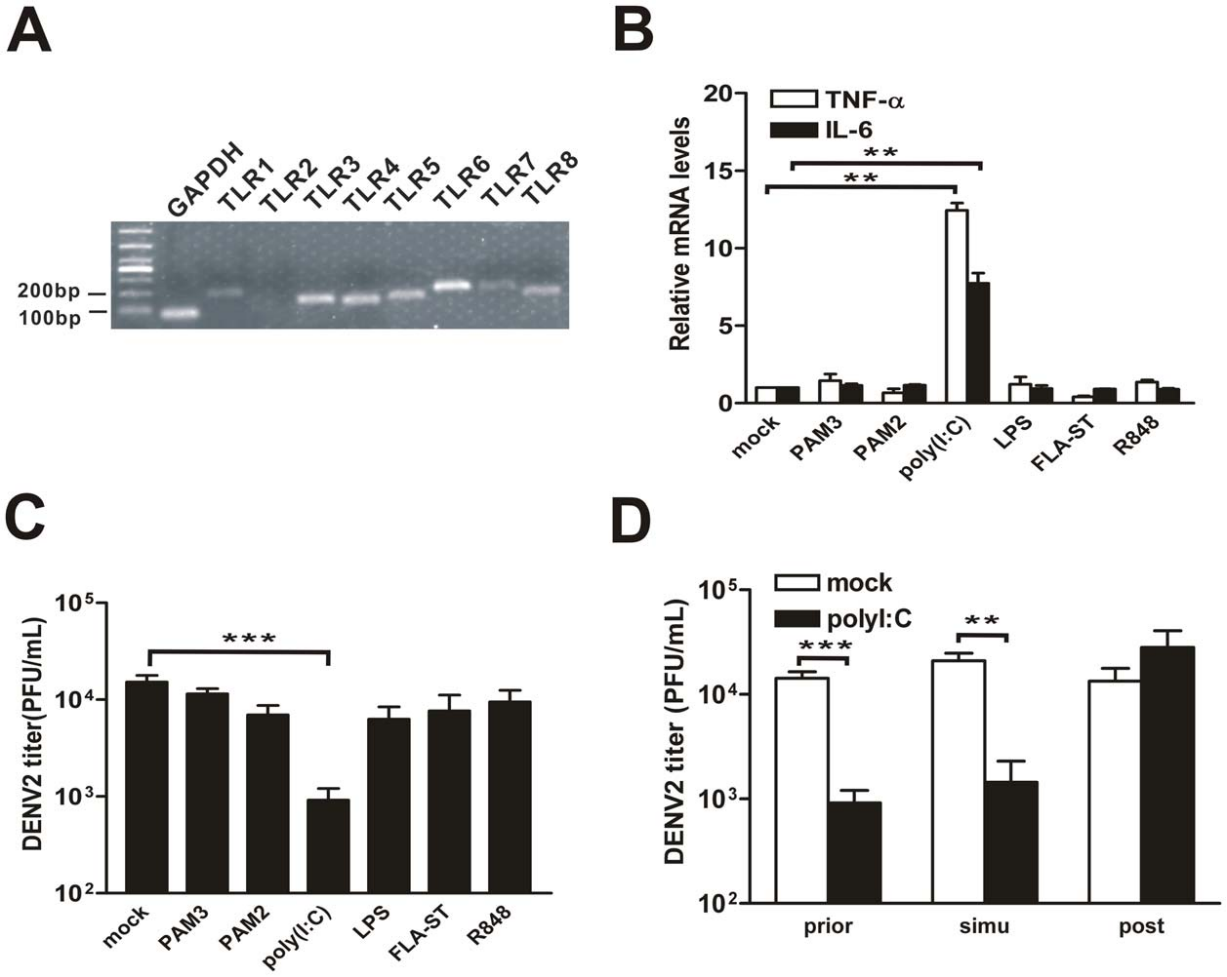
Effect of TLR ligands on DENV2 replication in HepG2 cells. (A) Endogenous TLR expression in HepG2 cells. (B) Induction of IL-6 and TNF-α in response to TLR ligands. HepG2 cells were pretreated with individual TLR ligands for 9 h. (C) Extracellular virus yields. HepG2 cells were pretreated with individual TLR ligands and infected by DENV2 for 24 h. (D) Extracellular virus yields. HepG2 cells were pretreated with poly(I∶C) at 9 h prior to, simultaneously (simu), or 9 h post DENV2 infection. Supernatants were harvested at 24 h p.i. and titered on C6/36 cells. Error bars represent the standard error of mean from the average of three experiments. Student's *t* test, **, p<0.01; ***, p<0.001.

Previous study have shown that TLRs, including TLR2, TLR3, TLR6, TLR9, are consistently expressed in HepG2 cells, while TLR1, TLR4, TLR5, TLR8 are expressed at a very weak level [28]. To validate the expression of TLRs in HepG2 cells, conventional RT-PCR was carried out. The result showed that expression of most TLRs, including TLR1 through TLR8 (except for TLR2), were detected (Fig. 1A). To further determine whether TLR signaling pathway is activated, the induction of proinflammatory cytokine IL-6 and TNF-α in each TLR ligands pretreated HepG2 cells were measured by real-time PCR. Surprisingly, only treatment of TLR3 ligand significant induced both IL-6 and TNF-α in HepG2 cells, but not other TLR ligands, including PAM3 (TLR1/2), PAM2 (TLR2/6), LPS (TLR4), FLA-ST(TLR5) or R848 (TLR7/8) (Fig. 1B).

To further test whether treatment of TLR ligands has an effect in the DENV2 replication, HepG2 cells were treated with individual TLR ligands followed by DENV2 infection. And the extracellular virus yields in infected cells were examined by TCID50 assay. As shown in Fig. 1A, about 10^4^ PFU/ml viral particles were produced in mock treated cells, confirming that HepG2 cells are permissive to DENV. Among the TLR ligands, TLR3 ligand poly(I∶C) pretreatment significantly decreased the DENV2 yields (by 90%), while all the other TLR ligands, including ligands of TLR1/6, TLR2/6, TLR4, TLR5 or TLR7/8 had no or very weak effect on the DENV2 production.

The extracellular DENV2 yields were also tested among three groups of HepG2 cells that were challenged with poly(I∶C) at different time points, namely 9 h prior to, simultaneously, or 9 h post virus inoculation. Our data showed that simultaneous treatment of poly(I∶C) with DENV2 infection significantly decreased the production of virus particles by 90% (Fig. 1B). By contrast, post-treatment of poly(I∶C) totally failed to execute a protective effect, resulting in a similar virus amount as non-treated control.

### Pretreatment of poly(I∶C) suppressed the DENV2 replication

**Figure 2:**
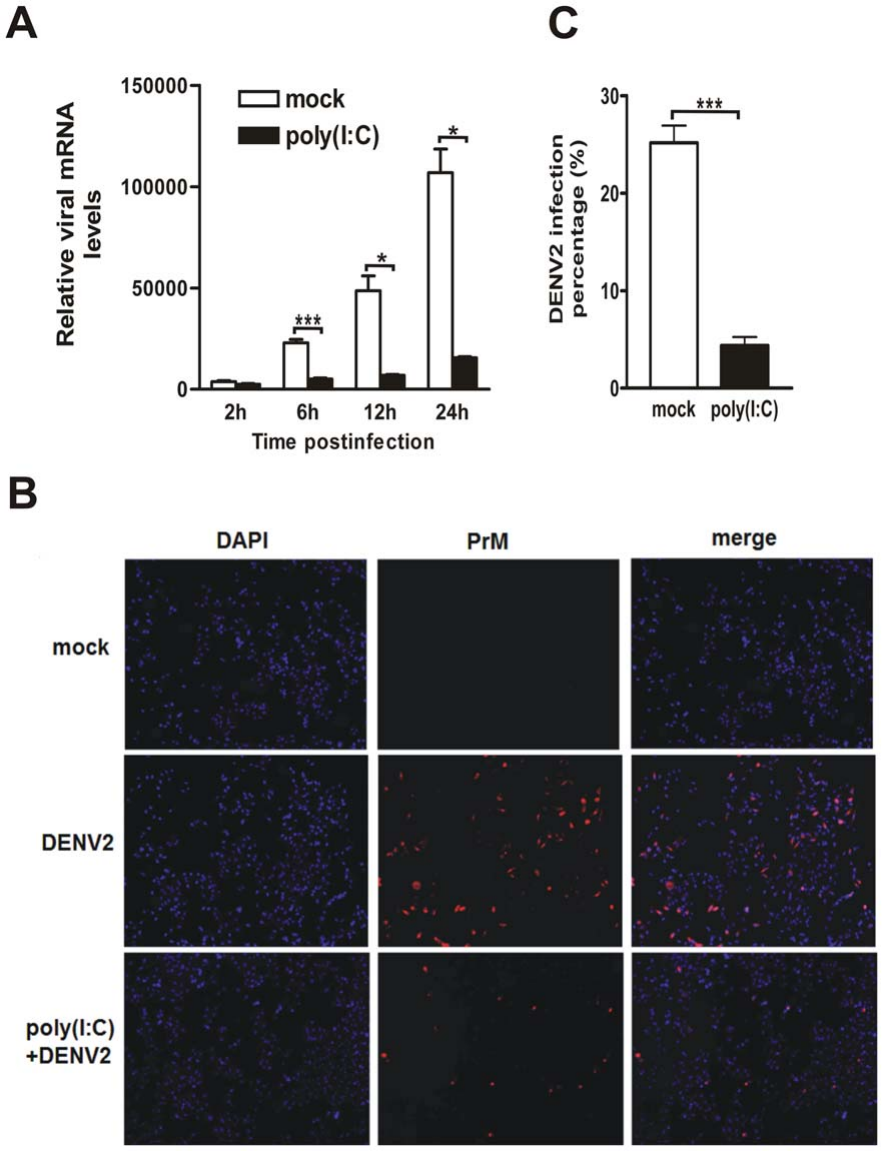
Pretreatment of poly(I∶C) suppresses the DENV2 replication in HepG2 cells. HepG2 cells were mock- or pretreated with 5 µg poly (I∶C) for 9 h, then infected with DENV2. (A) Real-time PCR to measure viral mRNA levels. Total RNAs were harvested at 2, 6, 12, and 24 h p.i., and used for real-time RT-PCR. (B) Immunofluorescence microscopy. Infected cells were fixed at 24 h p.i. and incubated with DENV2 prM antibody. (C) Percentages of positive-stained cells determined by PicCnt 100×. Error bars represent the standard error of mean from the average of three experiments. Student's *t* test, *, p<0.05; ***, p<0.001.

Because pre- and simultaneous treatment with poly(I∶C) resulted in a similar anti-DENV2 effect, the poly(I∶C) pretreatment was hence chosen for all following investigations. To determine whether poly(I∶C) inhibits DENV2 production by blocking DENV2 replication at transcriptional or posttranscriptional level, the expression of viral mRNA or protein in poly(I∶C)-treated HepG2 cells have been tested. The kinetic viral mRNA levels of DENV2 were progressively increased in both control (white bars) and poly(I∶C) pretreated HepG2 cells (black bars) after virus infection at 6, 12 and 24 h p.i (Fig. 2A). However, the increasing extent of viral mRNA copies was drastically attenuated by 78% (6 h p.i.), 85% (12 h p.i.), and 85% (24 h p.i.) respectively, in poly(I∶C)- vs mock-pretreated cells (6 h: P<0.001; 12 h: P<0.05; 24 h: P<0.05). Moreover, the prM protein of DENV2 was examined by immunofluorescence microscopy using the anti-prM antibody. Approximate 25% HepG2 cells were prM positive at 24 h after infection with DENV2 virus, while there were only 4% cells positively stained in poly(I∶C) pretreated cells (p<0.001) (Fig. 2B, 2C). There was no positive staining in the mock-infected control cells.

### Pretreatment of poly(I∶C) induced type I and III IFN expression

**Figure 3:**
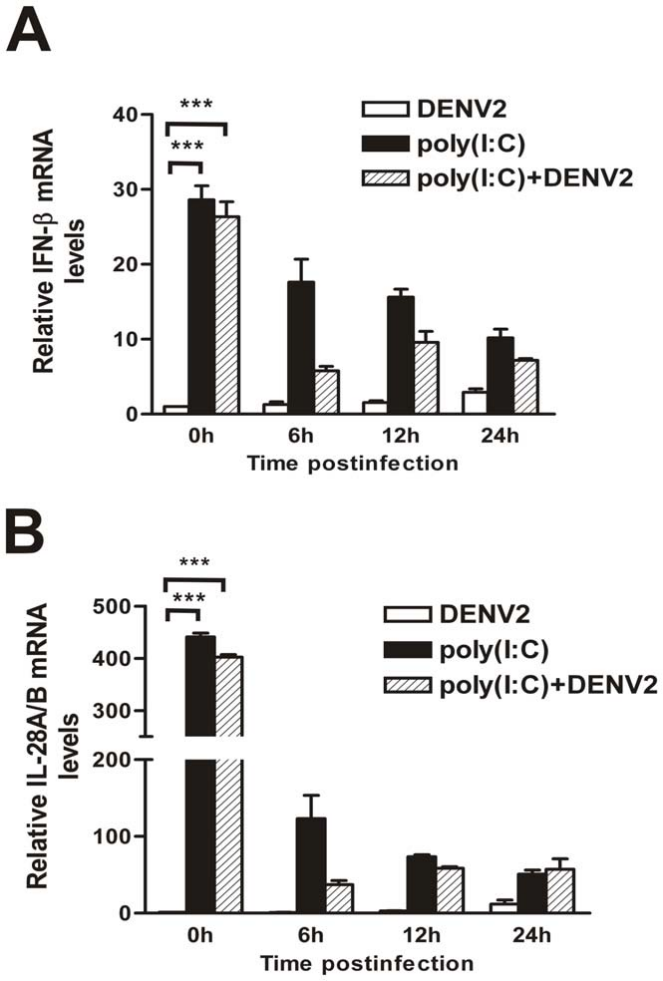
Expression of IFNs in mock-treated or poly(I∶C) pretreated HepG2 cells. After pretreatment with poly(I∶C) for 9 h, HepG2 cells were infected with DENV2 at an MOI 1. The cells were harvested at 0, 6, 12, and 24 h p.i. for RNA extraction and real-time RT-PCR for IFN-β (A) or IL-28A/B (B). Error bars represent the standard error of mean from the average of three experiments. Student's *t* test, **, p<0.01; ***, p<0.001.

To investigate the mechanism by which poly(I∶C) pretreatment inhibits the DENV2 replication, expression of IFNs, including type I IFN (represented by IFN-β), type II (IFN-γ) and type III (IL-28A/B) were examined by real-time PCR. IFN-γ expression was not altered by poly(I∶C) treatment (data not shown). However, the expression levels of IFN-β and IL-28A/B were significantly increased by 30- or 400- fold respectively (P<0.001) (0 h p.i., Fig. 3A, 3B). Moreover, the IFN-β and IL-28A/B mRNA expression levels gradually decreased after removal of poly(I∶C) (6, 12, and 24 h p.i., Fig. 3A, 3B, black bars). The infection of DENV2 did not elicit an additional induction of IFN-β and IL-28A/B within 24 h p.i., in either mock- or poly(I∶C)- treated HepG2 cells (Fig. 3A, 3B, white bars and hatched bars).

### Poly(I∶C)-inhibited virus replication was mediated through IFN-β

**Figure 4:**
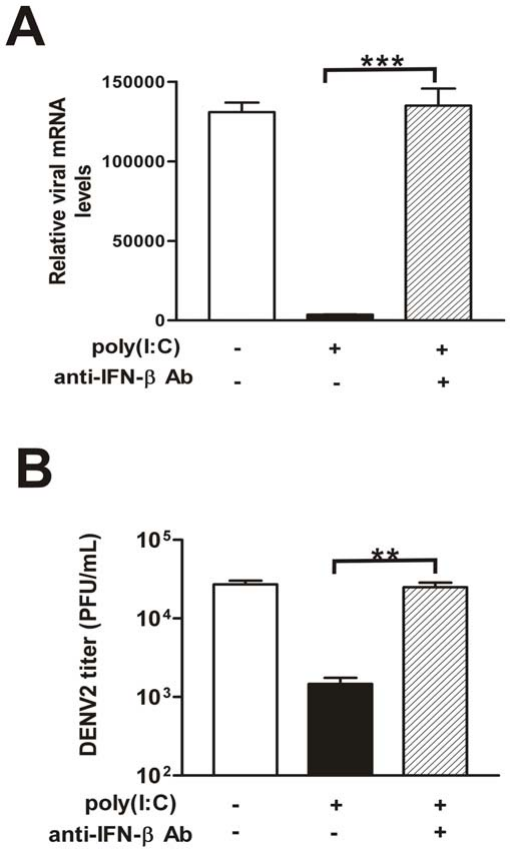
DENV2 replication levels in IFN-β neutralizing antibody treated HepG2 cells. 500 unit/ml IFN-β neutralizing antibody was added at 1 h before poly(I∶C) treatment and kept through poly(I∶C) treatment and virus infection. (A) Real-time PCR detecting the expression of viral mRNA; (B) Extracellular viral production determined by TCID50. Supernatants were harvested at 24 h p.i. and titered on C6/36 cells. Error bars represent the standard error of mean from the average of three experiments. Student's *t* test, **, p<0.01; ***, p<0.001.

Although the expression of IL-28A/B was induced by poly(I∶C) treatment, pretreatment of IL-28A/B alone was not able to effectively protect HepG2 cells from DENV2 infection (data not shown). To test the role of IFN-β, the viral replication levels in the presence of a neutralizing antibody against IFN-β were examined. Surprisingly, the neutralization of IFN-β only was sufficient to restore the viral replication. In Fig. 4A and 4B, the viral mRNA levels in poly(I∶C) pretreated cells and the extracelluar virus yields (hatched bars) were completely restored by the IFN-β neutralizing antibody, reaching a similar level to DENV2 infection alone (white bars). The viral replication levels recovered by neutralizing antibody were about 17-fold higher than poly(I∶C) pretreated cells (black bars).

### Poly(I∶C)-mediated IFN production and virus replication suppression were dependent on IKK

**Figure 5:**
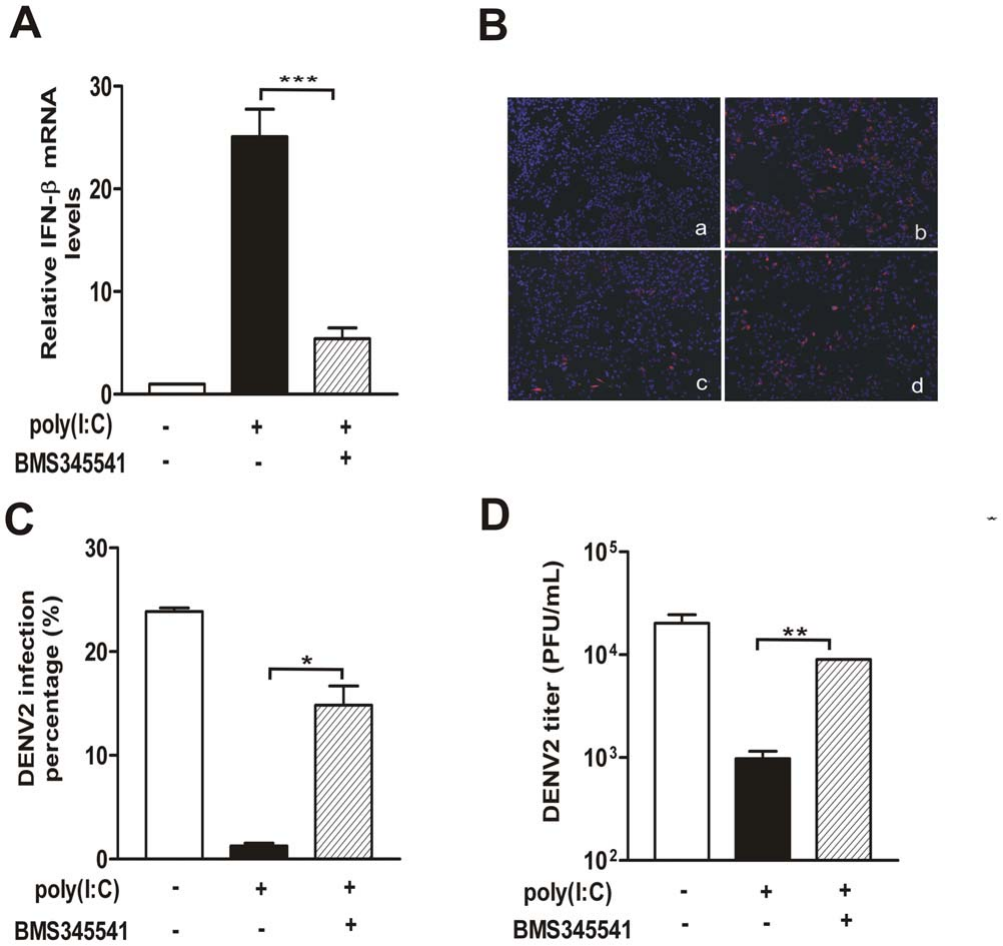
IKK inhibitor reversed the IFN-β induction and virus replication suppression mediated by poly(I∶C) pretreatment. HepG2 cells were incubated in control or IKK inhibitor for 30 min before poly(I∶C) pretreatment and virus infection. (A) Cells were harvested for measurement of IFN-β expression level; (B) Immunofluorescence microscopy. Cells were fixed at 24 h p.i. and incubated with DENV2 prM antibody. Panel a, mock infected; b, DENV2; c, DMSO+poly(I∶C)+DENV2; d, BMS345541+poly(I∶C)+DENV2; (C) Percentages of positive-stained cells determined by PicCnt 100×; (D) Extracellular viral production determined by TCID50. Supernatants were harvested at 24 h p.i. and titered on C6/36 cells. Error bars represent the standard error of mean from the average of three experiments. Student's *t* test, *, p<0.05; **, p<0.01; ***, p<0.001.

To further establish the co-relationship between the IFN-β and DENV2 replication mediated by poly(I∶C), we compared the IFN-β expression and viral replication levels in the cells treated with an IKK inhibitor (BMS-345541) before poly(I∶C) treatment and DENV2 infection. In the presence of IKK inhibitor (hatched bars, Fig. 5A), induction of IFN-β by poly(I∶C) was significantly decreased by 78.5% compared to poly(I∶C) pretreated cells (P<0.001)(black bars, Fig. 5A) as predicted. On the other hand, percentage of positive stained cells (approximate 15%) was significantly recovered by addition of IKK inhibitor (panel d, Fig. 5B; hatched bar, Fig. 5C) compared to the nontreated control (panel c, Fig. 5B; black bar, Fig. 5C). Moreover, the attenuated extracellular virions (black bar, Fig. 5B) were also significantly recovered by IKK inhibitor (hatched bar, Fig. 5D), and it was only 50% less than non-treated control (white bar, Fig. 5D).

## Discussion

TLRs are an important family of PRRs to detect PAMPs of invading pathogen and initialize innate immune response. TLRs and their signaling pathways are emerging as novel targets of therapeutics [29]. In the present study, we screened the effects of TLRs in the DENV2 replication in a widely used DENV permissive cell line, hepatoma HepG2. We demonstrated that TLR3 ligand poly(I∶C) has a protective effect in DENV2 replication, in which IFN-β plays a determinant role.

Differential effects of TLR ligands in suppressing DENV2 replication were observed. Our PCR result found that most of TLRs, except for TLR2, are endogenously expressed in the HepG2 cells, which was slightly different from previous reports showing other TLRs, such as TLR1 and TLR8 are not or weakly expressed while TLR2 is expressed [14], [28]. The difference of TLR expression might be due to the different resources of HepG2 cells or the efficacy of PCR primers. Strikingly, despite of the presence of these TLRs, the IL-6 and TNF-α production were not induced by most TLR ligands (except for TLR3) treatment, indicating that these TLR signaling pathways were not activated by their ligands in HepG2 cells. The absence of TLR activation is probably a major reason that these TLR ligands fail to protect cells from DENV2 infection. Our data found that LPS (TLR4 ligand) pretreatment did not reduce the DENV infection in HepG2 cells, which agrees with previous work by Cabrera-Hernandez *et al.* [25], but differs from Chen's finding that LPS inhibited DENV infection by 1–2 log in TLR-abundant immune cells (primary monocytes/macrophage) [24]. These contradictory observations could be attributed to the different TLR4 abundances and activation extents in different experimental cells. Our *in vitro* result was further validated by a latest study showing that *in vivo* cocurrent activation of TLR3/7/8 decreased viremia and melioration of DENV1 infection via increased inflammatory and humoral responses [30].

Protective role of type I and II IFNs has been well established in inhibiting DENVs infection [5]. Our study showed that poly(I∶C) pretreatment led to an induction of type I and III IFNs in HepG2 cells, but not type II IFN, which is consistent with the fact that IFN-γ is majorly expressed in NK or activated T cells [3]. To investigate whether anti-DENV effect by TLR3 ligand is IFN-dependent, we measured the viral replication levels in the presence of IFN-β neutralizing antibody or IKK inhibitor. Our results demonstrated that IFN-β neutralizing antibody was sufficient to recover the viral replication suppressed by poly(I∶C), which indicates that IFN-β is the determinant factor in the TLR3 protective effect. To be noted, the poly(I∶C)-induced IFN-β expression was not totally abrogated by IKK inhibitor and the DENV2 infection was not fully recovered, most probably because IKK inhibitor used here is designed to effectively inhibit IKK-1, but not other IKKs, such as IKKε, a key regulatory protein in IFN signaling pathway via phosphorylating IRF-3 [3]. Apparently, the poly(I∶C) inhibitory mechanism in our model is distinct from one of LPS in monocytes or macrophages model, which directly interfered with the virus entry in a CD14-dependent manner [24].

Furthermore, our real-time PCR data showed that the viral mRNA levels were decreased by poly(I∶C) pretreatment, indicating that poly(I∶C) protective effect might be executed as early as viral transcription stage. It is well-known that antiviral effects of IFN-β are mediated by ISG proteins, which cooperate to combat virus replication at each step [3], [31]. Among these ISGs, 2′,5′-oligoadenylate synthetase (OAS) 1, together with its downstream effector RNase L, play an antiviral role at transcription level. OAS1 and RNase L inhibit a broad range of RNA viruses by degradation of viral RNA [32]. In a recent study, the infection of DENV2 PL046 strain was reduced by overexpression of OAS or RNase L, and enhanced by knockdown of RNase L in human cell lines [33]. In our assay, the OAS expression was upregulated by 10-fold with poly(I∶C) pretreatment (data not shown), suggesting that OAS and RNase L are, at least partially, responsible for the TLR3-mediated protective effect, while engagement of other ISGs, such as protein kinase regulated by dsRNA (PKR) and Mx1, could not be excluded [3], [31].

Our study was the first to detect an induction of type III IFNs in HepG2 cells in response to poly(I∶C) challenge. *In vitro*, type III IFNs were shown to have a broad antiviral activity against HBV, HCV and other viruses through a similar mechanism as type I IFNs [4], [34], [35], [36], [37], [38], [39]. Although we did not find a significant antiviral role of type III IFNs in DENV2 replication in HepG2 cells, the induction of type III IFNs might still be of other biologic significance *in vivo*. For example, Barlett *et al.* showed that IL-28A did not combat vaccinia virus *in vitro*, but had a potent antiviral and immunostimulatory activity in mice by increasing lymphocytes in bronchial alveolar lavages and CD4+ T cells in total-lung lymphocyte preparations [35]. Therefore, more in-depth and *in vivo* investigations on role of the type III IFN in DENV infection will further validate the clinical appliance of TLR3 ligands in the DENV diseases.

Finally, our data indicate that the time of poly(I∶C) treatment was crucial to establish an antiviral state. Significant antiviral effect was found in poly(I∶C) treatment prior to virus inoculation or simultaneously, but not post-treatment. Because IFNs and ISGs induced by TLR activation are rapidly synthesized in a few hours, cells could produce sufficient agents to block viral replication in the former two conditions. However, in the latter condition, the failure of poly(I∶C) to protect cells could be because viral antagonisms were synthesized even earlier than IFNs induced by poly(I∶C) which was applied at 9 h after viral infection [40], [41]. For an instance, viral NS4B blocks IFN signaling pathway by interfering the phosphorylation of STAT1. This result supported a previous conclusion that IFNs have no obvious effect on established DENV infection, although they inhibit DENV replication [5]. The time-dependent effect of TLR3 ligand suggests that, the potential application of TLR3 ligand in vaccines or vaccine adjuvants against DENV diseases is greater than in the curative drugs.

In summary, our work presented first evidence that a timely activation of TLR3 signaling protects HepG2 cells from DENV2 infection, majorly through induction of IFN-β. Effect of TLR3 on other serotype DENV replication and in other cells, as well as related molecular mechanisms, are still under investigation. In-depth illustrations of TLR3 protective mechanism will greatly contribute to its clinical appliance. Therefore, our study provided substantial evidence that poly(I∶C) is a promising immunomodulatory agent against DENV infection and is potential in the DENV vaccines.

## Materials and Methods

### Cell culture

Hepatoma cell line HepG2 (ATCC, HB-8065) [24] and mosquito cell line C6/36 (ATCC, CRL-1660) [42] were maintained in Dulbecco's modified Eagle's medium (DMEM) complemented with 5% or 10% (v/v) fetal bovine serum (Hyclone) respectively, 1% sodium pyruvate, 100 µg/ml of penicillin and 100 units/ml streptomycin (Invitrogen).

### Virus

The Dengue-2 virus New Guinea C strain was provided by Guangzhou Centers for Disease Control, and propagated in C6/36 cells. C6/36 cells were inoculated with DENV2 at a low multiplicity of infection (MOI), and incubated at 35°C for 48–96 h. The supernatants were collected and clarified by centrifugation (1000 g, 5 min). Viral concentrations were titered on C6/36 cells and viral stocks were stored at −80°C.

### Treatment of TLR ligands

HepG2 cells were treated with following TLR ligands: 1 µg/ml Pam3CSK4 (TLR1/2), 100 ng/ml Pam2CSK4 (TLR2/6), 5 µg/ml poly(I∶C) (TLR3), 1 µg/ml LPS (TLR4), 100 ng/ml *S. typhimurium* Flagellin (FLA-ST) (TLR5), or 5 µg/ml R848 (TLR7/8) for 9 h respectively, and DENV2 infection was carried out at an MOI of 1. All TLR ligands were purchased from Invivogen.

### RT-PCR

Total RNA was prepared from HepG2 cells using TRIzol reagent (Invitrogen) following manufacturer's instruction. Reverse transcription was carried out by using 1 µg of RNA (Invitrogen). The sequences and product size for each pair of PCR primers were listed in Table 1. IFN-γ primer was purchased from SABiosciences. For conventional PCR, the reactions with HepG2 cDNA began with an initial denaturing cycle at 94°C for 1 min, followed by 32 cycles of 94°C for 40 s, 55°C for 40 s, 72°C for 1 min 15 s and a final extension step at 72°C for 5 min. For the real-time PCR, the reactions were performed in triplicate with each cDNA template by using IQ SYBR green Supermix (Bio-Rad) and performed on Bio-Rad CFX96 real-time Detection System (Bio-Rad).

**Table 1:** Primers for RT-PCR.

Gene Name	Sequence (5′-3′)	Product size(bp)
GAPDH	F:GCCTTCCGTGTCCCCACTG	72
	R:CGCCTGCTTCACCACCTTC	
TLR1	F:CCTACTGTGAACCTCAAGCAC	185
	R:TCTCCTAAGACCAGCAAGACC	
TLR2	F:TCACTCAGGAGCAGCAAGCA	129
	R:TGTGACATTCCGACACCGAGA	
TLR3	F:GATCTGTCTCATAATGGCTTGT	129
	R:GGCAAAGATATCCAGTTCTTCA	
TLR4	F:AGCCTAAGCCACCTCTCTACCT	116
	R:AGATTTGTCTCCACAGCCACCA	
TLR5	F:TGCCACTGTTGAGTGCAAGTC	132
	R:ACCTGGAGAAGCCGAAGGTAAG	
TLR6	F:TGTAGTGAGCTGAGACAGCG	181
	R:GTTCTCCTGAAGAGCAAGGAAG	
TLR7	F:AGCTTTAACCTCTCGCCATTACA	181
	R:TTGAGCAGAAGCCAACTTCACT	
TLR8	F:CTTCAGTCGTCAATGCTGACCT	139
	R:GATTGCTGCACTCTGCAATAACT	
IFN-β	F:AAACTCATGAGCAGTCTGCA	168
	R:AGGAGATCTTCAGTTTCGGAGG	
IL-28A/B	F:TTCACACCCTGCACCATATCCT	175
	R:AGGCGGAAGAGGTTGAAGGT	
DENV2-C Protein	F:TCCTAACAATCCCACCAACAGCA	137
	R:AGTTCTGCGTCTCCTGTTCAAGA	
IL-6	F:ATTCGGTACATCCTCGACGGCA	121
	R:CAGCCATCTTTGGAAGGTTCAGGT	

### Immunofluorescence microscopy

Cells were seeded onto coverslips (30–50% confluency) and infected with DENV2 (MOI 1) or left uninfected. Cells were fixed at 24 h after infection with 4% paraformaldehyde at 4°C for 30 min, and permeabilized with PBS containing 0.1% Triton X-100. Dengue virus was detected using a mouse antibody against DENV2 prM (1∶10, AbCam) and a secondary anti-mouse antibody (1∶1000, Invitrogen). Cell nuclei were stained with DAPI. Stained samples were visualized using Zeiss fluorescence microscope.

### Neutralizing assay

HepG2 cells were incubated with neutralizing anti-IFN-β (500 U/ml, Calbiochem) for 1 h prior to treatment with poly(I∶C). After 9 h, DENV2 infection was carried out at an MOI of 1. The media was replaced with fresh medium containing 500 U/ml neutralizing anti-IFN-β after DENV2 adsorption.

### IKK inhibition assay

HepG2 cells were incubated with 10 µM IKK inhibitor III (BMS-345541, Merck) for 30 min, followed by 9 h poly(I∶C) pretreatment and DENV2 infection.

### Virus titration

DENV2 titers in harvested supernatants were determined by TCID50 assay. Samples were serially diluted and inoculated into C6/36 cells in 96-well plates. After a 5-day incubation at 35°C and 5% CO2, cells were examined for cytopathic effects (CPE) under a light microscope. The virus titer (TCID50/ml) was calculated using the Reed–Muench method [43]. And 1 TCID50/ml was equivalent to 0.69 PFU/ml [44].

### Statistic Analysis

An unpaired, two-tailed Student's *t* test was used to determine the significance of real-time RT-PCR, Immunofluorescence microscopy and TCID50 assay. Data were considered significant at p<0.05.

## References

[1] Halstead SB (2007). Dengue.. Lancet.

[2] Gubler DJ (1998). Dengue and dengue hemorrhagic fever.. Clin Microbiol Rev.

[3] Borden EC, Sen GC, Uze G, Silverman RH, Ransohoff RM (2007). Interferons at age 50: past, current and future impact on biomedicine.. Nat Rev Drug Discov.

[4] Ank N, West H, Paludan SR (2006). IFN-lambda: novel antiviral cytokines.. J Interferon Cytokine Res.

[5] Diamond MS, Roberts TG, Edgil D, Lu B, Ernst J (2000). Modulation of Dengue virus infection in human cells by alpha, beta, and gamma interferons.. J Virol.

[6] Johnson AJ, Roehrig JT (1999). New mouse model for dengue virus vaccine testing.. J Virol.

[7] Rodriguez-Madoz JR, Bernal-Rubio D, Kaminski D, Boyd K, Fernandez-Sesma A (2010). Dengue virus inhibits the production of type I interferon in primary human dendritic cells.. J Virol.

[8] Tsai YT, Chang SY, Lee CN, Kao CL (2009). Human TLR3 recognizes dengue virus and modulates viral replication in vitro.. Cell Microbiol.

[9] Qin CF, Zhao H, Liu ZY, Jiang T, Deng YQ (2010). Retinoic acid inducible gene-I and melanoma differentiation-associated gene 5 are induced but not essential for dengue virus induced type I interferon response.. Mol Biol Rep.

[10] Nasirudeen AM, Wong HH, Thien P, Xu S, Lam KP (2011). RIG-I, MDA5 and TLR3 synergistically play an important role in restriction of dengue virus infection.. PLoS Negl Trop Dis.

[11] Uematsu S, Akira S (2007). Toll-like receptors and Type I interferons.. J Biol Chem.

[12] Blasius AL, Beutler B (2010). Intracellular toll-like receptors.. Immunity.

[13] Wu MH, Zhang P, Huang X (2010). Toll-like receptors in innate immunity and infectious diseases.. Front Med China.

[14] Xia C, Lu M, Zhang Z, Meng Z, Zhang Z (2008). TLRs antiviral effect on hepatitis B virus in HepG2 cells.. J Appl Microbiol.

[15] Wu J, Lu M, Meng Z, Trippler M, Broering R (2007). Toll-like receptor-mediated control of HBV replication by nonparenchymal liver cells in mice.. Hepatology.

[16] Isogawa M, Robek MD, Furuichi Y, Chisari FV (2005). Toll-like receptor signaling inhibits hepatitis B virus replication in vivo.. J Virol.

[17] Svensson A, Bellner L, Magnusson M, Eriksson K (2007). Role of IFN-alpha/beta signaling in the prevention of genital herpes virus type 2 infection.. J Reprod Immunol.

[18] Nazli A, Yao XD, Smieja M, Rosenthal KL, Ashkar AA (2009). Differential induction of innate anti-viral responses by TLR ligands against Herpes simplex virus, type 2, infection in primary genital epithelium of women.. Antiviral Res.

[19] Trapp S, Derby NR, Singer R, Shaw A, Williams VG (2009). Double-stranded RNA analog poly(I∶C) inhibits human immunodeficiency virus amplification in dendritic cells via type I interferon-mediated activation of APOBEC3G.. J Virol.

[20] Zhou Y, Wang X, Liu M, Hu Q, Song L (2010). A critical function of toll-like receptor-3 in the induction of anti-human immunodeficiency virus activities in macrophages.. Immunology.

[21] Wang N, Liang Y, Devaraj S, Wang J, Lemon SM (2009). Toll-like receptor 3 mediates establishment of an antiviral state against hepatitis C virus in hepatoma cells.. J Virol.

[22] Lau YF, Tang LH, Ooi EE, Subbarao K (2010). Activation of the innate immune system provides broad-spectrum protection against influenza A viruses with pandemic potential in mice.. Virology.

[23] Boukhvalova MS, Sotomayor TB, Point RC, Pletneva LM, Prince GA (2009). Activation of interferon response through toll-like receptor 3 impacts viral pathogenesis and pulmonary toll-like receptor expression during respiratory syncytial virus and influenza infections in the cotton rat Sigmodon hispidus model.. J Interferon Cytokine Res.

[24] Chen YC, Wang SY, King CC (1999). Bacterial lipopolysaccharide inhibits dengue virus infection of primary human monocytes/macrophages by blockade of virus entry via a CD14-dependent mechanism.. J Virol.

[25] Cabrera-Hernandez A, Thepparit C, Suksanpaisan L, Smith DR (2007). Dengue virus entry into liver (HepG2) cells is independent of hsp90 and hsp70.. J Med Virol.

[26] Modhiran N, Kalayanarooj S, Ubol S (2011). Subversion of innate defenses by the interplay between DENV and pre-existing enhancing antibodies: TLRs signaling collapse.. PLoS Negl Trop Dis.

[27] Thepparit C, Phoolcharoen W, Suksanpaisan L, Smith DR (2004). Internalization and propagation of the dengue virus in human hepatoma (HepG2) cells.. Intervirology.

[28] Nishimura M, Naito S (2005). Tissue-specific mRNA expression profiles of human toll-like receptors and related genes.. Biol Pharm Bull.

[29] Zhu J, Mohan C (2010). Toll-like receptor signaling pathways–therapeutic opportunities.. Mediators Inflamm.

[30] Sariol CA, Martinez MI, Rivera F, Rodriguez IV, Pantoja P (2011). Decreased Dengue Replication and an Increased Anti-viral Humoral Response with the use of Combined Toll-Like Receptor 3 and 7/8 Agonists in Macaques.. PLoS One.

[31] Samuel CE (2001). Antiviral actions of interferons.. Clin Microbiol Rev.

[32] Silverman RH (2007). Viral encounters with 2′,5′-oligoadenylate synthetase and RNase L during the interferon antiviral response.. J Virol.

[33] Lin RJ, Yu HP, Chang BL, Tang WC, Liao CL (2009). Distinct antiviral roles for human 2′,5′-oligoadenylate synthetase family members against dengue virus infection.. J Immunol.

[34] Ank N, West H, Bartholdy C, Eriksson K, Thomsen AR (2006). Lambda interferon (IFN-lambda), a type III IFN, is induced by viruses and IFNs and displays potent antiviral activity against select virus infections in vivo.. J Virol.

[35] Bartlett NW, Buttigieg K, Kotenko SV, Smith GL (2005). Murine interferon lambdas (type III interferons) exhibit potent antiviral activity in vivo in a poxvirus infection model.. J Gen Virol.

[36] Kotenko SV, Gallagher G, Baurin VV, Lewis-Antes A, Shen M (2003). IFN-lambdas mediate antiviral protection through a distinct class II cytokine receptor complex.. Nat Immunol.

[37] Sheppard P, Kindsvogel W, Xu W, Henderson K, Schlutsmeyer S (2003). IL-28, IL-29 and their class II cytokine receptor IL-28R.. Nat Immunol.

[38] Robek MD, Boyd BS, Chisari FV (2005). Lambda interferon inhibits hepatitis B and C virus replication.. J Virol.

[39] Pagliaccetti NE, Chu EN, Bolen CR, Kleinstein SH, Robek MD (2010). Lambda and alpha interferons inhibit hepatitis B virus replication through a common molecular mechanism but with different in vivo activities.. Virology.

[40] Munoz-Jordan JL (2009). Subversion of interferon by dengue virus.. Curr Top Microbiol Immunol.

[41] Conceicao TM, El-Bacha T, Villas-Boas CS, Coello G, Ramirez J (2010). Gene expression analysis during dengue virus infection in HepG2 cells reveals virus control of innate immune response.. J Infect.

[42] Zhou JM, Tang YX, Fang DY, Zhou JJ, Liang Y (2006). Secreted expression and purification of dengue 2 virus full-length nonstructural glycoprotein NS1 in *Pichia pastoris*.. Virus Genes.

[43] Reed LJ, Muench H (1938). A simple method of estimating fifty percent endpoints.. The American Journal of Hygiene.

[44] Maul A (1991). Aspects statistiques des méthodes de quantification en virologie.. Virologie des milieux hydriques.

